# Optimizing personalized psychological well-being interventions through digital phenotyping: results from a randomized non-clinical trial

**DOI:** 10.3389/fpsyg.2024.1479269

**Published:** 2025-01-06

**Authors:** Giulia Rocchi, Emanuela Vocaj, Simone Moawad, Alessandro Antonucci, Carlo Grigioni, Vincenzo Giuffrida, Joy Bordini

**Affiliations:** ^1^Department of Dynamic, Clinical Psychology and Health, Faculty of Medicine and Psychology, Sapienza University of Rome, Rome, Italy; ^2^GoHealhty & Co Sagl, Lugano, Switzerland; ^3^Dalle Molle Institute for Artificial Intelligence Research, Lugano, Switzerland

**Keywords:** mobile assessment, EMAS, digital phenotyping, mental health, smartphone, unsupervised learning

## Abstract

**Background:**

Digital technologies, including smartphones, hold great promise for expanding mental health services and improving access to care. Digital phenotyping, which involves the collection of behavioral and physiological data using smartphones, offers a novel way to understand and monitor mental health. This study examines the feasibility of a psychological well-being program using a telegram-integrated chatbot for digital phenotyping.

**Methods:**

A one-month randomized non-clinical trial was conducted with 81 young adults aged 18–35 from Italy and the canton of Ticino, a region in southern Switzerland. Participants were randomized to an experimental group that interacted with a chatbot, or to a control group that received general information on psychological well-being. The chatbot collected real-time data on participants’ well-being such as user-chatbot interactions, responses to exercises, and emotional and behavioral metrics. A clustering algorithm created a user profile and content recommendation system to provide personalized exercises based on users’ responses.

**Results:**

Four distinct clusters of participants emerged, based on factors such as online alerts, social media use, insomnia, attention and energy levels. Participants in the experimental group reported improvements in well-being and found the personalized exercises, recommended by the clustering algorithm useful.

**Conclusion:**

The study demonstrates the feasibility of a digital phenotyping-based well-being program using a chatbot. Despite limitations such as a small sample size and short study duration, the findings suggest that digital phenotyping and personalized recommendation systems could improve mental health care. Future research should include larger samples and longer follow-up periods to validate these findings and explore clinical applications.

## 1 Introduction

In recent years, a growing body of research highlighted the importance of digital technologies, such as smartphones, social media and mobile applications, as promising approaches to expanding the availability of mental health services and improving access to care (Naslund and Aschbrenner, 2021; Schueller and Torous, 2020; Senbekov et al., 2020).

Investing in ‘digital mental health’ means taking an approach that harnesses the power of digital health technologies to mitigate the medical and socio-economic effects of mental illness (Lokmic-Tomkins et al., 2023). These technologies include mobile health applications (mHealth), wearable devices, consumer neurotechnologies, virtual reality systems, online platforms, and care coordination systems (Lu et al., 2020).

The growth of mobile applications for mental health is evidenced by the presence of approximately 380,000 apps available in the App Store and Play Store, with many downloads indicating a strong interest on the part of the population (ECHAlliance, s.d.). Various investigations suggest the popularity of mental health apps as an alternative tool for seeking help, facilitating self care and for avoiding social taboos and stigma related to seeking help for mental health support (Anderson et al., 2016; Kenny et al., 2016). Recent studies have shown that these apps, when integrated with psychotherapy pathways, are effective for a wide range of psychological disorders, including depression, bipolar disorder, schizophrenia, anxiety disorders and substance abuse disorders (Miralles et al., 2020; Torous et al., 2021). Smartphones and other wearable devices, such as smartwatches, enable the passive and uninterrupted collection of physiological and behavioral data due to their non-invasiveness and pervasiveness (Harrison et al., 2020). This process, known as *digital phenotyping*, refers to the “moment-to-moment quantification of the human phenotype at the individual level *in situ* using data from personal digital devices, particularly smartphones” (Onnela and Rauch, 2016; Torous et al., 2016). It is important to point out that digital phenotyping includes both passive and data related to mobile approaches including active monitoring of subjective experiences by means of ecological momentary assessments (EMAs), which involve repetitive sampling of subjects’ current behaviors and moods at different times of the day and metadata which regard the user’s interaction with an app (Marciano et al., 2023; Shiffman, 2009; Wilkinson et al., 2016).

Passive data collection through digital devices allows for the capture of ‘real-world’ behavior without the burden of frequent surveys or telephone assessments, making the data less influenced by the *Hawthorne effect*, i.e., behavior change in response to observation and assessment (Sedgwick and Greenwood, 2015). The integration of digital technologies into clinical practice can lead to a significant improvement in quality and access to care, and in revolutionizing the way we monitor and treat mental disorders (Marciano et al., 2023).

Overall, the use of Digital Phenotyping through a mobile approach seems to be feasible for various populations, including individuals with and without psychopathological symptoms, allowing for continuous and contextual assessment of mental health problems (Bufano et al., 2023; Oudin et al., 2023). Passive data collection through digital phenotyping allows the system to continuously monitor users’ behavioral and physiological patterns, providing a detailed and dynamic overview of their psychological states (Marengo and Montag, 2020; Moura et al., 2023). Passive data alone does not directly lead to symptom improvement, but it serves as a foundational component for delivering targeted therapeutic interventions. By combining passive data with advanced analytical algorithms, a mobile health app or a chatbot could generate tailored recommendations that address the specific needs of each individual for instance personalized therapeutic strategies, such as Cognitive Behavioral Therapy (CBT) or adaptive support interventions. For example, a user exhibiting irregular sleep patterns coupled with elevated stress levels might receive customized suggestions for stress management or targeted behavioral modifications. These personalized recommendations, when integrated into a structured therapeutic framework, have the potential to improve psychological well-being and contribute to positive clinical outcomes (Ferrari et al., 2022; Müller-Bardorff et al., 2024).

The main goal of this study was to investigate the feasibility of a psychological well-being program through a chatbot based on digital phenotyping, using a non-clinical trial. The decision to focus on psychological well-being as an index of our program’s effectiveness stems from the fact that, according to several reviews, intervention programs using smartphones have been able to detect usable markers of psychological well-being components such as life satisfaction, or happiness on a continuum (Rhim et al., 2020). As proven by several studies this data can be integrated into behavior change interventions to promote users’ health and well-being (Choi et al., 2024; Cornet and Holden, 2018; Lee et al., 2023).

This approach aims to evaluate how passive data collection via digital devices can be effectively implemented in mental health treatments (i.e., psychological assessments, psychotherapy, support group, peer support) to monitor psychological distress, prevent psychological malaises and improve the well-being of users. A further goal of the study was the creation of a user profiling and content recommendation system. Using the smartphone data collected through digital phenotyping and the active participation of users, the recommendation system we implemented, was designed to analyse the behavioral and physiological patterns of users, enabling personalized and targeted recommendations to improve psychological well-being of users.

## 2 Method

*Ad hoc* for this project, a chatbot integrated into the Telegram application, available for Android and iOS users, was developed in collaboration with the start-up Go Healthy & Co Sagl. The chatbot was used to assess the participants’ psychological well-being and deliver the intervention.

### 2.1 Participants

The study involved a one-month randomized non-clinical trial. Participants were 81 young adults aged 18–35 years (M = 26.2; SD = 7.92), recruited in Italy and in the Canton of Ticino, an Italian-speaking region of southern Switzerland. Inclusion criteria were a good knowledge of Italian and no diagnosis of mental disorders. Participants were recruited via online advertisements through social media such as Facebook and Instagram. Randomization into experimental or control groups was carried out by means of a randomization algorithm included in the initial form. Participants in this study expressed their willingness to participate in the study by signing the informed consent and received no compensation for their participation. The study received approval from the Ethics Committee of Università della Svizzera italiana (protocol No.CE_2022_12) and complies with the Declaration of Helsinki of the World Medical Association (1964).

### 2.2 Stages of the non-clinical trial

As a preliminary step, all the participants completed a baseline questionnaire to collect socio-demographic information, current well-being, malaise, information on behavioral addictions, and the presence of a mental health diagnosis or current psychological treatment. Participants with a diagnosis of mental health problems or undergoing psychological treatment were excluded. Prior to the start of data collection, two focus groups were conducted for a preliminary test on the usability of the application.

### 2.3 Interventions and evaluations

Participants in the experimental group have access to a Telegram chatbot for 30 days designed to collect passive data through sensors embedded in users’ smartphones along with EMAs on their emotional state and well-being, to collect active data from the participants, coherently with the definition of digital phenotyping. Every day, participants received a message from the chatbot asking: “How do you feel now?” Participants could answer the question via a Likert scale from 0 (*very bad*) to 10 (*very good*).

The interactions with the chatbot were based on a decision tree whose branching is driven by the participants’ answers. Each decision/answer determines the next step, aiming to understand the user’s psychological state better. When a leaf of such a decision tree is reached, the interaction ends, and an *exercise* integrated into the application is proposed to the participant to improve her/his mood or promote well-being.

The chatbot sent a notification message twice a day to collect *in situ* information on participants’ well-being or discomfort. During such interactions, ourchatbot aimed to identify underlying emotions and suggested *exercises* based on positive psychology and cognitive-behavioral therapy, selected from psychological manuals and research articles reporting their effectiveness (Bendig et al., 2019; Gabrielli et al., 2021).

The exercises aimed to raise users’ awareness of their well-being or discomfort and suggest coping behaviors. In this study, we consider 17 different exercises. The users’ responses activate various *natural language processing* (NLP) models, including intention identification, emotion and polarity classifiers (Liu, 2020). At the end of each exercise, two feedbacks were requested from the users similar to the initial questions. The first, related to the well-being situation, was the question: “Overall, how satisfied are you with your life nowadays?” received an answer based on a value from 0 (*not at all*) to 5 (very satisfied). The same question was asked at the end of the program. The second feedback was focused on the potential usefulness of the exercise performed by the participant where the question “Was the exercise useful?” Received an answer based on a value from 0 (*not at all useful*) to 4 (very useful). For the duration of the non-clinical trial, the feature of providing emergency numbers for local mental health services in extreme cases was introduced. Participants in the control group, after giving informed consent and completing the baseline questionnaire, received an online brochure with general information on mental health. There was no interaction with the chatbot in this group.

### 2.4 Data collection

Ourchatbot collects the following information through digital phenotyping and EMAs:

- the entire conversation between the user and chatbot;
- the users’ responses for each step of the decision tree;
- the users’ responses on the usefulness of the exercise;
- the type of exercise provided and completed; and
- the time of each interaction with the chatbot.

### 2.5 Training a recommender system: methodological aspects

Besides investigating the feasibility of a psychological well-being program based on digital phenotyping, the trial’s goal was also to evaluate the potential of *machine learning* tools to more flexibly assign the exercises to the participants based on their interaction with the chatbot, also taking into account the information collected during the preliminary step.

### 2.6 A recommender system for big data

Many *supervised* learning methods (e.g., regressions) could be used to train a fitness function able to assign scores to the exercises depending on the specific values of the features describing a patient (Murphy, 2022). This requires annotated data, which experts could, at least in principle, provide. Yet, here, we cope with so-called *big data*, meaning that the number of features (130 for our study) vastly exceeds the number of data points (i.e., the patients, thus 81). Applying a *feature selection* does not change the situation, as the descriptive features considered by the study cover very different characteristics and behaviors of the participants. Any direct supervised approach is expected to perform poorly for such a setup (Zhou et al., 2017).

We therefore consider a different approach involving unsupervised methods (Ghahramani, 2003). In practice, we apply a *clustering* algorithm to the feature vectors associated with the different patients. More specifically, we adopt distance-based techniques, where a metric function describing the dissimilarity between two (instances of the features describing the) participants is required. The clustering algorithm forms clusters of participants by minimizing the intra-cluster distances and maximizing the inter-cluster ones. Instead of basic *hard* clustering algorithms, which sharply assign a single cluster to the instance of the features associated with a participant, we adopt *soft* clustering tools (Murphy, 2022), where the membership in a cluster is expressed by means of a probabilistic value.

This is the key idea to cope with the (big) data from the trial and solve the exercise recommendation task. To train the recommender system in a supervised fashion, we describe the participant with their memberships to the different clusters, whose number is considerably smaller than the number of features, thus mitigating the problem due to the scarcity of training data. Once the clusters are formed, we approach the recommendation task at the cluster level by simply comparing, separately for each exercise, the distribution over the usefulness values reported for that particular exercise by all the users and the distribution associated with the users of a specific cluster. This allows us to simply characterize the average fit of an exercise for a cluster as the normalized deviation between the means of the two distributions.

### 2.7 Data preprocessing

From a machine learning perspective, the data should first be converted into a tabular structure with a row for each participant and a possibly large collection of descriptive features. Then, all the non-numerical data should be converted into numerical ones to be processed by machine learning algorithms. For features represented by categorical variables, this required a simple *one-hot encoding* approach where, as an example, the three values (*female*, *male* and *non-binary*) of the categorical feature *gender*, were converted into three Boolean variables taking values zero or one in an exhaustive and exclusive way. For features that are already numerical we discretise according to the quantiles of the empirical distribution. E.g., for the *age* of the participants, we have five groups based on the ranges 16–19, 20–22, 23–25, 26–32, and 33+. Text interactions have been instead processed by NLP tools to translate them to numerical features (Mikolov et al., 2013). As, for some of those interactions, the number of texts was not constant among the participants, we also performed aggregations and averages in order to give the data a perfect tabular structure with numerical values only. All the features have been finally scaled between zero and one. This is important for the specification of the *similarity measure* between two participants we need for the clustering (Sect. 3.1). To this aim, we consider a weighted *Manhattan distance* (Faisal et al., 2020). The scaling prevents biases between the features in the clustering algorithm. In contrast, the weights associated with the different features are manually specified by the experts to model the relative importance of the different features. This also includes an expert-based additional *feature selection* automatically achieved when the experts set a zero weight for a particular feature.

### 2.8 Clustering algorithm implementation

Instead of adopting probabilistic clustering algorithms (Murphy, 2022), which natively achieve soft clustering, the need for distance-based clustering motivated us to adopt the popular *K-means* algorithm (Hartigan and Wong, 1979). However, the distance from the cluster centroids can be assumed to be inversely proportional to the probability of that object belonging to a particular cluster. K-means receives the required number of clusters as an input. The optimal value for this number can be decided by the *silhouette method* and agreed upon with the domain experts (Wang et al., 2017). In our application, both approaches led to coping with four clusters. The iterative procedure of K-means was tested with different sets of features and different sets of weights provided by different experts for the distances. Notably, almost identical clusters have been obtained. The clustering results, including cluster assignments and centroids, were saved for further analysis. To understand the feature importance for each cluster, SHAP (SHapley Additive exPlanations, Lundberg, 2017) values were computed. SHAP is a popular tool that adds explainability to machine learning algorithms. The tool is based on a game-theoretic interpretation of the contributions of the model features. A fast computation of the Shapley values associated with the predictions related to different “coalitions” of features. Here, the method is adapted to a clustering task. These SHAP values helped identify the most influential features for each cluster.

### 2.9 Recommender system implementation

Let us detail how we select the exercises to be recommended to a *test* participant, i.e., a participant whose descriptive features are not included in the trial database. The recommendation system starts by calculating the distances of each (non-test) participant from the centroids of the four clusters. These distances are crucial as they represent how closely a profile matches each cluster’s characteristics. Those distances are then used to weigh the recommendations for the different exercises. To convert the distances into relative weights for the clusters, a negative exponential smoothing function is applied. This function transforms the distances so closer clusters have higher weights, reflecting a stronger similarity to the user’s profile. For each exercise, the utility values for each participant are aggregated based on their cluster assignment. The exercise’s average utility for participants in each cluster is computed and compared to the overall mean utility across all participants. The standard deviation of the utility values normalizes the difference between the cluster-specific average utility and the overall mean utility. This normalized difference is then weighted by the importance of the cluster for the test participant. The scores for each exercise are renormalized by the sum of the weights, resulting in the final expected utility for each exercise. The exercises are then ranked based on their expected utilities, with higher expected utilities indicating more relevant and beneficial exercises for the user. This ranking helps in identifying the most suitable exercises to recommend to each user, tailored to their unique profile and cluster assignment.

## 3 Results

**Figure 1:**
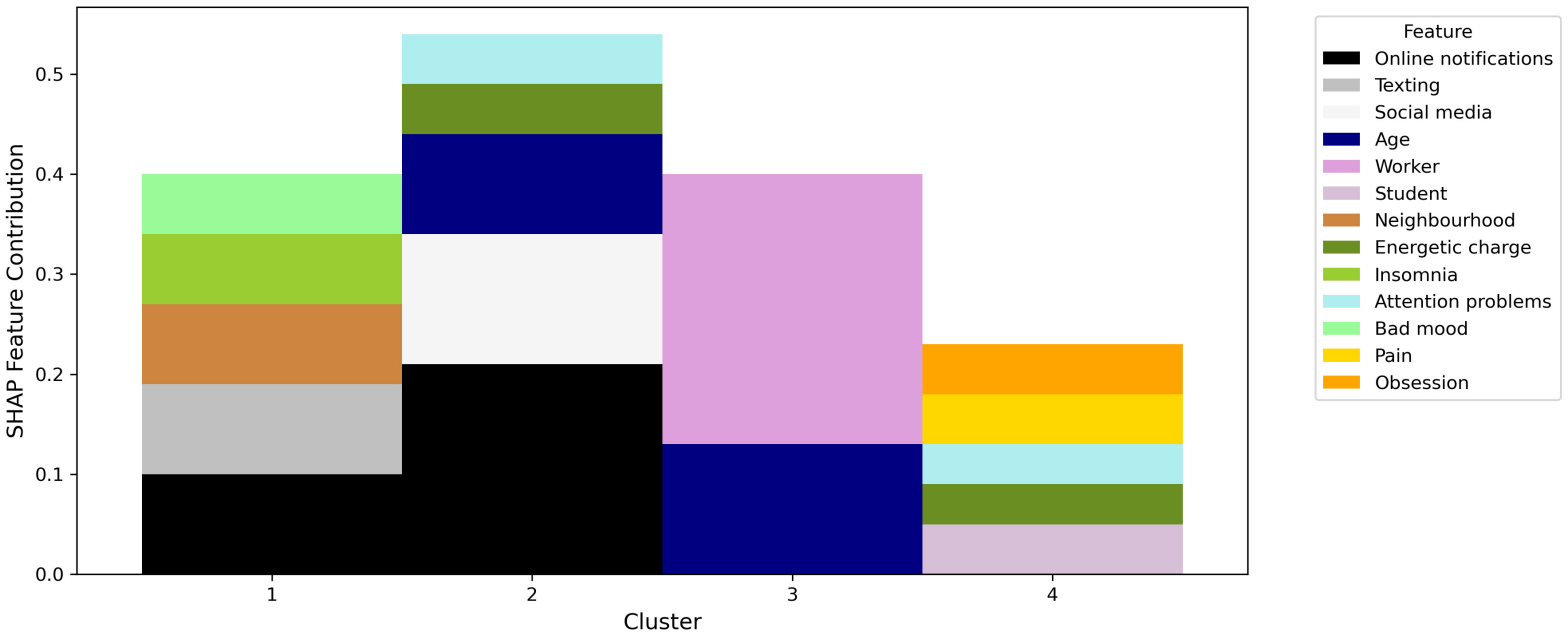
SHAP (SHapley Additive exPlanations) feature contributions for the four identified clusters, highlighting the most influential features in each cluster.

Our study involved 81 young adults (62.1% female, 35.6% male and 2.3% rather not answered), 60.9% of the participants resided in Italy and 39.1% in the Canton of Ticino in Switzerland. The majority of our participants were students (59.8%) with at least a high school degree (48.3%). Finally, most of the participants indicated their socio-economic status as ‘moderately good’ (40.2%). At the time of onboarding, on a scale between one and five, the well-being of the participants was on average 3.06, by the end of the program the average well-being of participants was on average 4.1. The average score with respect to the usefulness of the exercises proposed to the participants was 3.77. Additionally, SHAP summary plots were generated to visualize the contributions of different features to the clustering results. The clustering analysis revealed four distinct clusters with balanced cardinalities. Table 1 presents detailed summaries of each cluster, including the number of participants and the most influential features based on SHAP values. Figure 1 presents a visual summary of the study, with each cluster’s four most relevant features (apart from Cluster 3, which has only two features, as we do not depict the features whose SHAP value is less than 0.04 for the sake of readability). For example, the contribution strength of the feature “worker” in Cluster 3 is 0.27. This means that if a participant had identical features but was a student instead of a worker, their probability of belonging to Cluster 3 would decrease by 27%.

**Table 1:** Summary of key features for each cluster, based on the SHAP values.

Cluster	1	2	3	4
Participants	19	16	24	22
Feature #1	Online notifications (5.00)	Online notifications (3.88)	Worker (2.50)	Obsession (1.25)
Feature #2	Texting (1.00)	Social media (2.63)	Age (3.13)	Student (2.48)
Feature #3	Neighborhood (1.28)	Age (1.73)	//	Pain (2.65)
Feature #4	Insomnia (3.11)	Attention problems (2.43)	//	Attention problems (2.32)
Feature #5	Bad mood (2.84)	Energetic charge (2.01)	//	Energetic charge (2.02)

The numeric values between parentheses are the cluster centroid values for the specific features. Ranges are between one and five.

The frequency of online notifications and texting activities are the most present features for participants in Cluster 1. Individuals in this cluster report higher levels of insomnia and general unease. Participants in Cluster 2 digital phenotype is characterized by the high frequency of online notifications and the use. The presence of these factors is consistent with their difficulties in maintaining attention and energy levels in life. Participants in Cluster 3 are primarily influenced by their occupation and age. Participants in Cluster 4 are mostly students reporting obsessive thoughts, and experience of pain. They also report difficulties in maintaining attention and energy levels in life. In Figure 1 the *y*-axis represents the SHAP feature contribution values, while the *x*-axis lists the cluster numbers (1–4). Each cluster displays a stacked bar chart where the contribution of each feature is color-coded according to the legend on the right.

## 4 Discussion

The integration of digital technologies into clinical practice represents a fundamental step toward a more personalized and accessible approach to mental health care (Schueller and Torous, 2020; Torous et al., 2021). This study explored the feasibility of a psychological non-clinical trial based on digital phenotyping, using a chatbot to collect passive and active data from participants’ smartphones. The results provide several relevant implications for clinical practice and future research.

Our study’s results indicate that a chatbot based on the construct of digital phenotyping integrated into a popular messaging application was effective in monitoring psychological well-being. Previous researches demonstrated that the chatbot’s ability to collect active and passive real-time data and provide personalized recommendations based on users’ behavioral and physiological patterns shows significant potential in reducing the burden on healthcare providers and improving access to mental health services (Miralles et al., 2020; Naslund and Aschbrenner, 2021). Furthermore, the positive feedback from participants regarding the usefulness of the exercises proposed by the chatbot suggests that such interventions can be useful for enhancing psychological well-being as shown in literature (Bufano et al., 2023; Choi et al., 2024).

The identification of four distinct clusters based on the data collected through digital phenotyping allows for the creation of a more personalized user profiling and content recommendation system. By understanding the specific needs and responses of each cluster, we can deliver more targeted recommendations to improve psychological well-being. This approach aligns with the study’s aim of leveraging digital phenotyping for better mental health interventions.

### 4.1 Study limitations and future directions

Despite the promising results, this study has some limitations. First, the relatively short duration of the non-clinical study (1 month) and the follow-up limited to 4 months may not be sufficient to fully evaluate the long-term effectiveness of the proposed interventions. Future studies should consider larger and more diverse samples and longer follow-up periods to validate the results obtained. In addition, the small sample, limited to young adults without diagnosed mental disorders, may not be representative of the general population. Finally, as we took into account a population that self-reported as non-clinical, no clinical scales were included to measure any psychological distress conditions. The only psychological variable that was investigated was that of general well-being through a self-assessment. For future studies, it would be interesting to explore the application of digital phenotyping and personalized recommendation systems in clinical populations with diagnosed mental disorders. Therefore, the integration of more data from wearable devices and other digital data sources could further enrich the predictive power and effectiveness of tailored interventions.

## 5 Conclusion

This study demonstrated the feasibility and potential of a psychological well-being program based on digital phenotyping, using a chatbot to collect data and deliver personalized interventions. The results suggest that digital technologies can improve access to mental health care, providing innovative tools for continuous monitoring and intervention. Despite some limitations, the evidence suggests that the approach of digital phenotyping and personalized recommendation systems could represent an innovation in mental health care. Further research is needed to explore the application of these technologies in broader and more diverse clinical contexts. Indeed, it is widely recognized in the scientific literature that this type of study needs further clinical validation taking into account several factors including: type of clinical population examined, cultural context, digital methodologies used and general biases including the placebo effect (Hauser et al., 2022; Onnela, 2021). Collaboration between researchers, technology developers and mental health professionals will be critical to developing innovative and integrated solutions that can improve access and quality of mental health care.

## Data Availability

The raw data supporting the conclusions of this article will be made available by the authors, without undue reservation.
